# Advances in the genome-wide association study of chronic hepatitis B susceptibility in Asian population

**DOI:** 10.1186/s40001-017-0288-3

**Published:** 2017-12-28

**Authors:** Bing Qiu, Wei Jiang, Mojtaba Olyaee, Kenji Shimura, Akihiro Miyakawa, Huijing Hu, Yongcui Zhu, Lixin Tang

**Affiliations:** 1 Department of Gastroenterology, Heilongjiang Province Hospital, 82 Zhongshan Road, Harbin, 150036 Heilongjiang People’s Republic of China; 2 Department of Gastroenterology, The First Affiliated Hospital of Jiamusi University, Jiamusi, 154002 People’s Republic of China; 3 Division of Gastroenterology, Department of Internal Medicine, University of Kansas, Medical Center, Kansas City, 66160 USA; 4 Department of Gastroenterology, Asahi General Hospital, Chiba, 289-2511 Japan; 5 Department of Laboratory Diagnosis, Heilongjiang Province Hospital, Harbin, 150036 People’s Republic of China

**Keywords:** Genome-wide association study, Chronic hepatitis B, Pathogenesis, Susceptibility

## Abstract

Chronic hepatitis B (CHB) is the most common chronic liver disease resulting from viral infection and has become a serious threat to human health. Each year, about 1.2 million people in the world die from diseases caused by chronic infection of hepatitis B virus. The genetic polymorphism is significantly associated with the susceptibility to chronic hepatitis B. Genome-wide association study was recently developed and has become an important tool to detect susceptibility genes of CHB. To date, a number of CHB-associated susceptibility loci and regions have been identified by scientists over the world. To clearly understand the role of susceptibility loci in the occurrence of CHB is important for the early diagnosis and prevention of CHB.

## Background

Chronic hepatitis B (CHB) is the most common chronic liver disease caused by viral infection and has now become a serious threat to human health. The number of chronic HBV-infected patients worldwide has so far risen to 350–400 million, of which 25 to 40% eventually died of liver cirrhosis and liver cancer [1–3]. In China, there is a high incidence of hepatitis B with more than 120 million carriers of HBV. Each year, nearly 300,000 HBV carriers die of liver cirrhosis and liver cancer [4]. The causes of CHB are complicated, among which the main risk factors include familial spread, infant infection, infection due to immunologic inadequacy, and a history of other liver diseases. With advances in the epidemiological study and molecular biology research, scientists have found that many gene polymorphisms are actually associated with CHB occurrence [5], which has greatly enriched our understanding in the occurrence and progression of CHB. In recent years, the rapid development of genome-wide association study (GWAS) has become an important tool to search for CHB-related susceptibility genes. GWAS has been applied to screen CHB-related candidate SNPs by using multiple approaches of molecular biology and bioinformatics. The combined effects of important CHB susceptibility loci and the interaction between gene and environment have been further investigated in-depth. To clearly understand the role of each susceptibility loci in the occurrence of CHB will provide a solid theoretical base for early diagnosis and prevention of CHB.

## Genome-wide association study

With the completion of Human Genome Project, GWAS became possible and developed rapidly. GWAS was designed to screen the disease-related genetic markers across a complete set of genomic DNA or genome to scrutinize genetic variations or single nucleotide polymorphisms (SNPs) associated with complex diseases. The database of genotypes and phenotypes (dbGAP), an international resource sharing center, was then established to archive and distribute data from studies such as GWAS in which associations between genetic variations and human diseases had been identified. GWAS aims at determining genes and susceptibility loci related to the occurrence of diseases, searching for genetic markers for the disease, therefore conducting early diagnosis and effective individualized treatment, as well as developing new drugs and novel specified disease prevention. GWAS has two types of design: single-stage design and two- or multi-stage design. Single-stage design is used to genotype SNP loci in a one-time manner for all selected case–control samples, and then analyze the association between each SNP and the disease. Two- or multi-stage design is a multi-step process used to genotype all SNP loci on a genome-wide scale in a small sample of population, followed by the selection of a few SNP loci found to be most significantly associated with the studied disease after statistical analysis. In another or multiple other larger independent populations, those selected SNPs will be further genotyped, and then analyzed according to the data obtained from the previous two or multi-stage studies. Single-stage design of GWAS is a costly process due to the large sample size required for genome-wide SNP genotyping. To reduce the number of genotyping and cost, most of investigators prefer to use a two- or multi-stage design. The main advantage of GWAS lies in its ability to comprehensively assess the association between genetic variations and diseases on a genome-wide scale without any biological hypothesis prior to the study.

Several methods such as DNA microarray analysis, exome sequencing, whole-genome sequencing were used while performing GWAS. DNA microarray (or DNA chip) is an important adjunct in performing GWAS because it allows a single sample to be simultaneously screened for variants at ~ 2 million known genetic markers in a single standardized assay at the whole-genome level. However, the high-throughput nature of DNA microarray combined with tremendous amount of datasets may result in a high rate of errors [6]. The reliability and reproducibility of microarrays are generally not satisfactory, which can be resulted from a number of factors such as the complexity of human genome, the quality of DNA prepared, the specificity of probes designed, the sample size, and the methods used for statistical analysis. To improve the reliability and reproducibility of microarray to some extent, all procedures involved should be tightly regulated and stringently quality controlled. Currently, microarray-based genotyping platforms were not widely used in GWAS [7]. Exome sequencing is a transcriptomics technique used to sequence all of the expressed genes in a genome (known as the exome). It is know that human exome only consists of 1% of the human genome, indicating the cost of sequencing can be substantially lowered in exome sequencing, typically only one-sixth to whole-genome sequencing. However, exome sequencing only covers 85% of disease-related variants and the remaining 15% of variants not located within the exon will escape from the screening [8]. Moreover, it is still not clear whether exome sequencing is able to fully capture genetic variants associated with complex disease [7]. Whole-genome sequencing has become cost and time efficient with the advent of next generation sequencing (NGS). Whole-genome sequencing provides greater coverage of the entire genome, which can capture both common and rare variants. However, despite the large drop in sequencing cost for a human genome, large-scale sequencing projects are still costly, which has prevented whole-genome sequencing from being widely used in GWAS that usually require thousands of samples [9]. The quality of the resulting draft sequence from whole-genome sequencing needs to be improved. Different NGS platforms used in GWAS may produce sequencing errors ranging from 0.5 to 2% [10]. Therefore, findings from whole-genome sequencing by using NGS should be further confirmed by more validated technologies.

## The application of GWAS to the study of disease susceptibility

Single nucleotide polymorphism (SNP) refers to a DNA sequence polymorphism at the genomic level, caused by only a single nucleotide variation that commonly occurs within a population. SNP is the most common genetic variation in human, accounting for over 90% of all known polymorphisms. In recent years, as the third generation of genetic markers, SNP has been widely used to search for disease-related genes and to explain the difference among individuals and groups in terms of the susceptibility to the disease and the disease progression [11]. Traditional analysis of the association between complex diseases and candidate genes usually selects one or a few disease-related candidate genes based on an understanding of the disease; therefore, genes and SNP loci for study are poorly available.

**Fig. 1:**
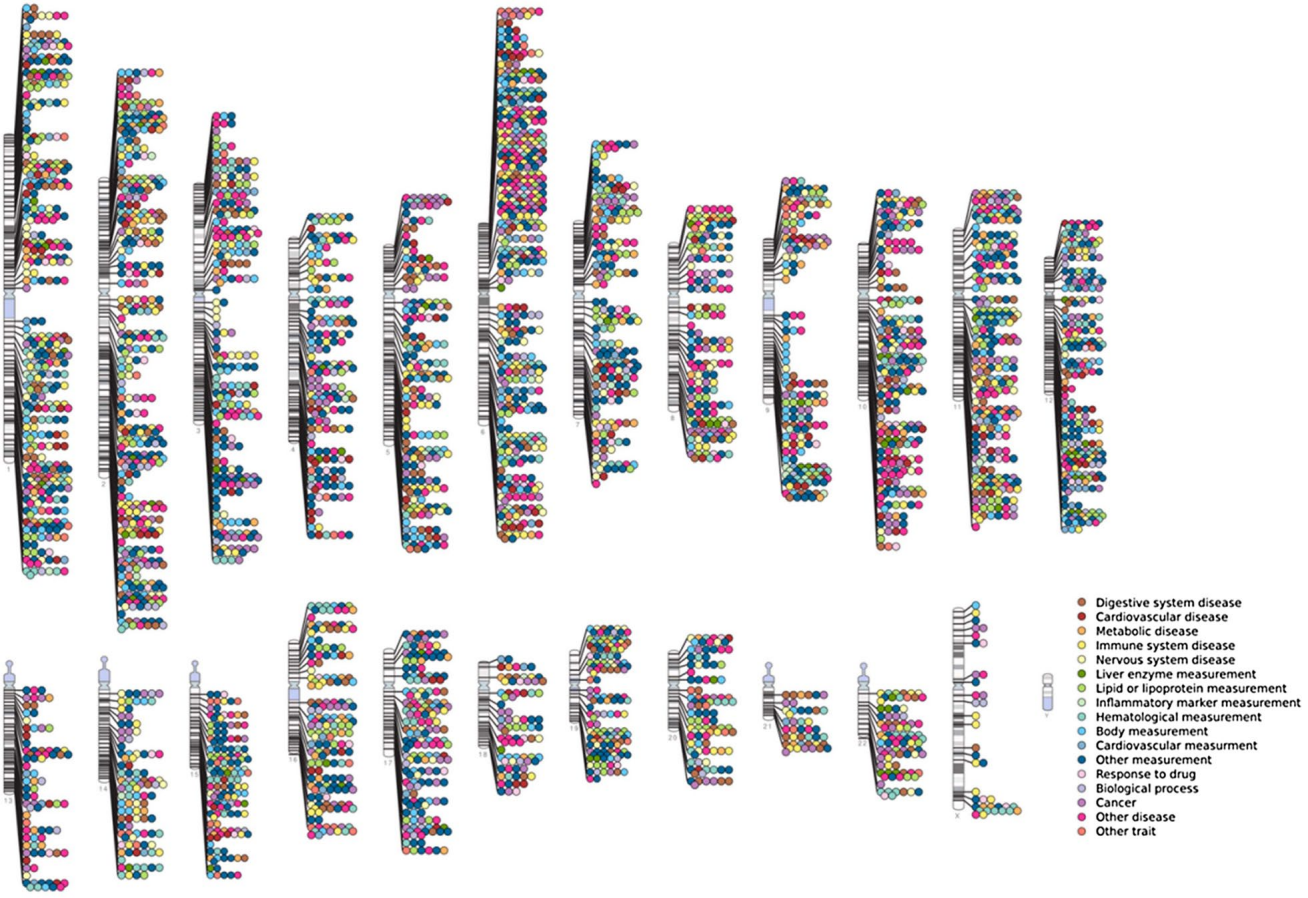
The chromosomal distribution of SNPs associated with complex diseases and traits (NHGRI GWA Catalog: http://www.genome.gov/GWAStudies)

In recent years, with the completion of two projects HapMap (The International HapMap Project) and 1000 Genomes (The 1000 Genomes Project) as well as the rapid development of high-throughput genotyping technology, GWAS has become an important tool for scientists to search for disease susceptibility-related genetic loci. In 2005, a GWAS for the age-related macular degeneration was first reported in *Science*, which is a hallmark for the application of GWAS to the medical field [12]. GWAS is generally performed to investigate millions of SNPs across the whole-genome, and is carried out on a large-scare, multi-center basis such that associations between diseases and genes can be repeated and confirmed. GWAS is not limited to a specific gene or chromosomal region, which offers itself a great potential to discover unknown genes related to certain disease under investigation. It also opens a new avenue to systematically study the genetic factors associated with complex diseases. Compared with traditional candidate gene study and family linkage study, GWAS presents to be much more effective in detecting disease-related genetic loci. Currently, GWAS has become a powerful tool to study the molecular etiology of complex diseases, and has greatly advanced the understanding of human diseases, such as macular retinae, breast cancer, prostate cancer, leukemia, coronary heart disease, obesity, diabetes, schizophrenia, and arthritis [13, 14]. These results have been published on many prestigious journals, in which a series of disease-related genes, genetic susceptibility regions and SNPs have been reported. As of December 2015, scientists have totally completed 1692 GWAS for 18 categories of nearly 300 complex diseases and traits, and have identified about 4000 SNPs that are associated with complex diseases and traits (Fig. 1).

## Application of GWAS to the study of CHB

The first GWAS on CHB was published in 2009. As of February 2016, a total of eleven GWAS on CHB have been reported. In these studies, a number of CHB-related susceptibility loci and genetic regions have been reported, among which 26 important candidate SNPs were identified (Table 1). As a newly emerging study tool, GWAS certainly needs to be further improved. The GWAS on CHB is recently getting started, therefore to summarize and properly interpret the GWAS data, and to understand the role of these loci in the pathogenesis of CHB in different populations will be critical for the application of GWAS to the study of CHB.

**Table 1:** GWAS on the genetic mechanism of chronic hepatitis B

	Ethnicity	SNP	Chromosome (locus)	Odds ratio	*P* value (combined stage)	References
1	Japanese Korean Indonesian	rs9277535	6 (HLA-DPB1)	1.39–1.89	3.0 × 10^−54^ −3.0 × 10^−12^	[10–12] [18, 19]
2	Japanese East Asian Korean	rs3077	6 (HLA-DPA1)	1.78–1.89	2.0 × 10^−61^ −2.0 × 10^−38^	[10–12] [14, 17, 18]
3	Japanese Korean	rs2856718	6 (TRNAI25)	1.56–1.60	4.0 × 10^−37^ 2.0 × 10^−24^	[11, 12] [17, 18]
4	Japanese Korean Chinese	rs7453920	6 (HLA-DQB2)	1.81–2.00	5.0 × 10^−37^ ~7.0 × 10^−26^	[11, 12, 16] [17, 18]
5	Korean	rs1419881	6 (TCF19)	1.37	1.0 × 10^−18^	[12, 18]
6	Korean	rs652888	6 (EHMT2)	1.38	7.0 × 10^−13^	[12, 18]
7	Korean	rs9394021	6 (VARS2)	0.78	1.7 × 10^−6^	[13]
8	Korean	rs2517459	6 (VARS2-SFTA2)	0.56	1.7 × 10^−10^	[13]
9	East Asian	rs9277542	6 (HLA-DPB1)	1.64	2.0 × 10^−21^	[14]
10	Chinese	rs11866328	16 (GRIN2A)	1.68	2.0 × 10^−8^	[15]
11	Chinese	rs4821116	22 (UBE2L3)	1.22	2.0 × 10^−12^	[16]
12	Chinese	rs3130542	6 (TRNAI25)	1.33	9.0 × 10^−14^	[16, 18]
13	Chinese	rs352140	3 (TLR9)	0.70	8.8 × 10^−3^	[17]
14	Chinese	rs16944	2 (IL1B)	0.67	1.6 × 10^−2^	[17]
15	Chinese	rs3212227	5 (IL12B)	1.38	2.1 × 10^−2^	[17]
16	Chinese	rs3799488	13 (IFNGR1)	1.48	4.8 × 10^−3^	[17]
17	Chinese	rs1059293	6 (IFNGR2)	0.27	1.1 × 10^−2^	[17]
18	Chinese	rs467960	21 (MX1)	0.68	2.2 × 10^−2^	[17]
19	Chinese	rs12614	6 (CFB)	1.89	1.28 × 10^−34^	[18]
20	Chinese	rs422951	6(NOTCH4)	1.27	5.33 × 10^−16^	[18]
21	Chinese	rs378352	6 (HLA-DOA)	1.26	1.04 × 10^−23^	[18]
22	Chinese	rs2853953	6 (HLA-C)	1.47	5.06 × 10^−20^	[18]
23	Chinese	rs1883832	20 (CD40)	1.21	2.95 × 10^−15^	[18]
24	Indonesian	rs9267665	6 (HLA)	2.05	1.0 × 10^−17^	[19]
25	Chinese	rs477515	6 (TRNAI25)	2.05	3.0 × 10^−19^	[20]
26	Indonesian Chinese	rs3135363	6 (HLA-DR)	1.53,1.51	7.0 × 10^−22^ 8.0 × 10^−7^	[19, 20]

Scientists and scholars at home and abroad have recently performed a series of GWAS on CHB and found multiple CHB-related SNPs. In 2009, Kamatani first reported the GWAS on CHB, in which he found two loci, rs9277535 within the HLA-DPB1 region and rs3077 within the HLA-DPA1 region, play important roles in the occurrence of CHB in Japanese population [15]. Following this study, Mbarek further confirmed Kamatani’s findings using GWAS also done with Japanese population [16]. Moreover, he and his colleagues found rs2856718-A within HLA-DQB1 region and rs7453920-G within HLA-DQB2 region are associated with the occurrence of CHB. In 2013, Kim [17] completed the GWAS on CHB using South Korea population, and found that rs652888 in the gene *EHMT2*, and rs1419881 in the gene *TCF19* within the HLA region, are associated with an increased risk of CHB occurrence. In addition, this group also verified that these loci-rs9277535, rs3077, rs7453920, and rs2856718 are all associated with the occurrence of CHB in both Japanese population and Korean population. Cheong et al. [18] recently found that rs9394021 and rs2517459 two SNP loci in the VARS2-SFTA2 gene region are the new genetic markers in the Korean population of CHB in 2015. Nishida N conducted the first GWAS on how to protect from hepatitis B and clear HBV [19]. It was found that rs3077 in HLA-DPA1 region and rs9277542 in HLA-DPB1 region, both have a protective effect on CHB in East Asian population, and both are associated with HBV clearance.

Chinese scholars Liu L et al. first conducted GWAS on CHB with Chinese Han population in 2011 and found that rs11866328 G, located in the *GRIN2A* gene within 16p13.2 region, is a susceptibility locus associated with disease progression in carriers of HBV [20]. Recently Hu Z and his colleagues found that rs3130542-A in the gene *TRNAI25* within 6p21.33 (near the HLA-C) region, and rs4821116-G in the gene *UBE2L3* within the 22q11.21 region, are both associated with the occurrence of CHB in Chinese Han population [21]. Moreover, they further confirmed that rs7453920 is associated with the occurrence of CHB in Japanese and Korean populations as previously reported; they also found that this locus is actually associated with high prevalence of CHB in Chinese Han population. in 2015, a GWAS study on CHB performed by He et al. [22] in southwest China revealed 6 novel loci associated with increased prevalence rate of chronic hepatitis B, which are rs352140 on the gene TLR9, rs16944 on the gene IL1B, rs3212227 on the gene IL12B, rs3799488 on the gene IFNGR1, rs1059293 on the gene IFNGR2, rs467960 on the gene MX1, respectively. In addition, they also confirmed that the previously discovered 4 SNP loci namely rs3077, rs2856718, rs9277535, and rs7453920, are associated with CHB in this area. In the same year, another GWAS study led by Jiang et al. [23] in the eastern part of China revealed 5 novel CHB susceptibility loci, including rs12614 on the gene CFB, rs422951 on the gene NOTCH4, rs378352 on the gene HLA-DOA, rs188332 on the gene HLA-C, and rs18838325 on the gene CD40, respectively. In 2016, Zhu et al. fine mapped the histocompatibility complex (MHC) region by using their existing GWAS data and identified four additional susceptibility loci that independently drove the chronic HBV infection in Han Chinese [24]. Nishida et al. applied HLA imputation method to determine HLA alleles by using genome-wide SNP typing data of 1975 Japanese individuals, and found that a SNP located in the HLA-DP locus from GWAS was strongly associated with CHB susceptibility [25]. Xiang et al. found that human leukocyte antigen DP/DQ gene (HLA-DP/DQ) polymorphisms (rs9277471, rs9277535, and rs9277542 in HLA-DP; rs9272346 in HLA-DQ) are associated with chronic hepatitis B in Chinese Han (400 patients) and Uygur (399 patients) populations [26]. In 2017, Shin et al. identified rs1265163 in OCT4 as a novel genetic marker for CHB susceptibility in a 3902 Korean individuals in a follow-up study to their GWAS [27].

In order to evaluate the immune response after injection of hepatitis B vaccine, Png E and his colleagues completed in 2011 the first GWAS on the immune response of hepatitis B vaccine in the population of Indonesia [28]. It was found that rs3135363 within the HLA-DR region, rs9277535 within the HLA-DPB1 region, and rs9267665 within the HLA-DP region are all associated with an immune response after the injection of hepatitis B vaccine. A subsequent GWAS completed by Pan also confirmed that rs3135363 is associated with an immune response after injection of hepatitis B vaccine in the Chinese Han population [29]. However, the locus rs477515-T located within the gene *TRNAI25*, 15 kb upstream of HLA-DRB1, showed no significant correlation with the immune response of hepatitis B vaccine.

The genetic background among human populations is generally different and the mechanism of pathogenesis can also differ. As a complex disease which involves multi-genes, chronic HBV infection is challenging to work on and the conclusions drew in the studies can be inconsistent due to the differences in the genetic background of populations and area studied. Therefore, the relationship between CHB and SNP loci screened by GWAS still remains unclear. More populations and larger independent samples are needed in order to improve the consistency of GWAS results.

## Conclusions

At present, GWAS only analyzes the effect of a single locus on the disease susceptibility. However, such effect on the complex disease is generally very weak, therefore this strategy cannot be simply taken to investigate the causes of complex diseases. Hence, how to find an effective way to perform in-depth analysis for GWAS data and then detect more susceptibility genes has become a new research hotspot [30]. To date, different strategies and approaches have been successively taken in the follow-up studies of GWAS for complex diseases in order to perform in-depth data mining. For example, when testing the correlation between a SNP and certain diseases, the interactions between this SNP and other SNPs will be also thoroughly examined (i.e., epistatic effect). Several analytical methods, such as Bayesian epistasis, classification and regression trees, and multifactor dimensionality reduction, have been widely used in data mining [31–33]. The application of these strategies and approaches has compensated for the disadvantage of classical GWAS, and has also deepened our understanding in the genetic mechanisms of complex human diseases. For this reason, the follow-up studies for the GWAS data are therefore of high importance.

## Abbreviations

CHB: chronic hepatitis B
HBV: hepatitis B virus
GWAS: genome-wide association study
